# Supplementary material for: Histone deacetylase 9 promoter hypomethylation associated with adipocyte dysfunction is a statin-related metabolic effect
Source: Clin Epigenetics. 2020 May 14;12:68. doi: 10.1186/s13148-020-00858-w (PMC7222462; doi:10.1186/s13148-020-00858-w)
Supplement: Supplementary file 1 — Additional file 1 – Supplementary Figures. Fig. S1: Differentiation of SGBS cell line. (a) Micorscopic mages of lipid droplets in pre-adipocytes (day 0) and differentiated mature adipocytes (day 12) visualised using microscopy (x10 magnification - scale bar 10 μM) (b) Expression of key adipose genes during a 12-day differentiation period (normalised to housekeeping gene B2M). For each gene, the fold-change compared to the day expression is first observed is presented. Data represent three biological replicates and plotted as the mean ± SEM. Fig. S2: All DMPs identified in response to a) mevastatin and b) atorvastatin treatment. Fig. S3: DMPs annotated to the promoter region that were also shared between mevastatin and atorvastatin treatments. Grey indicates log2 fold change < 1. Fig. S4: Differentially methylated region (DMR) in HDAC9 promoter shared between mevastatin and atorvastatin treatments. [file 13148_2020_858_MOESM1_ESM.pptx]

## Slide 1
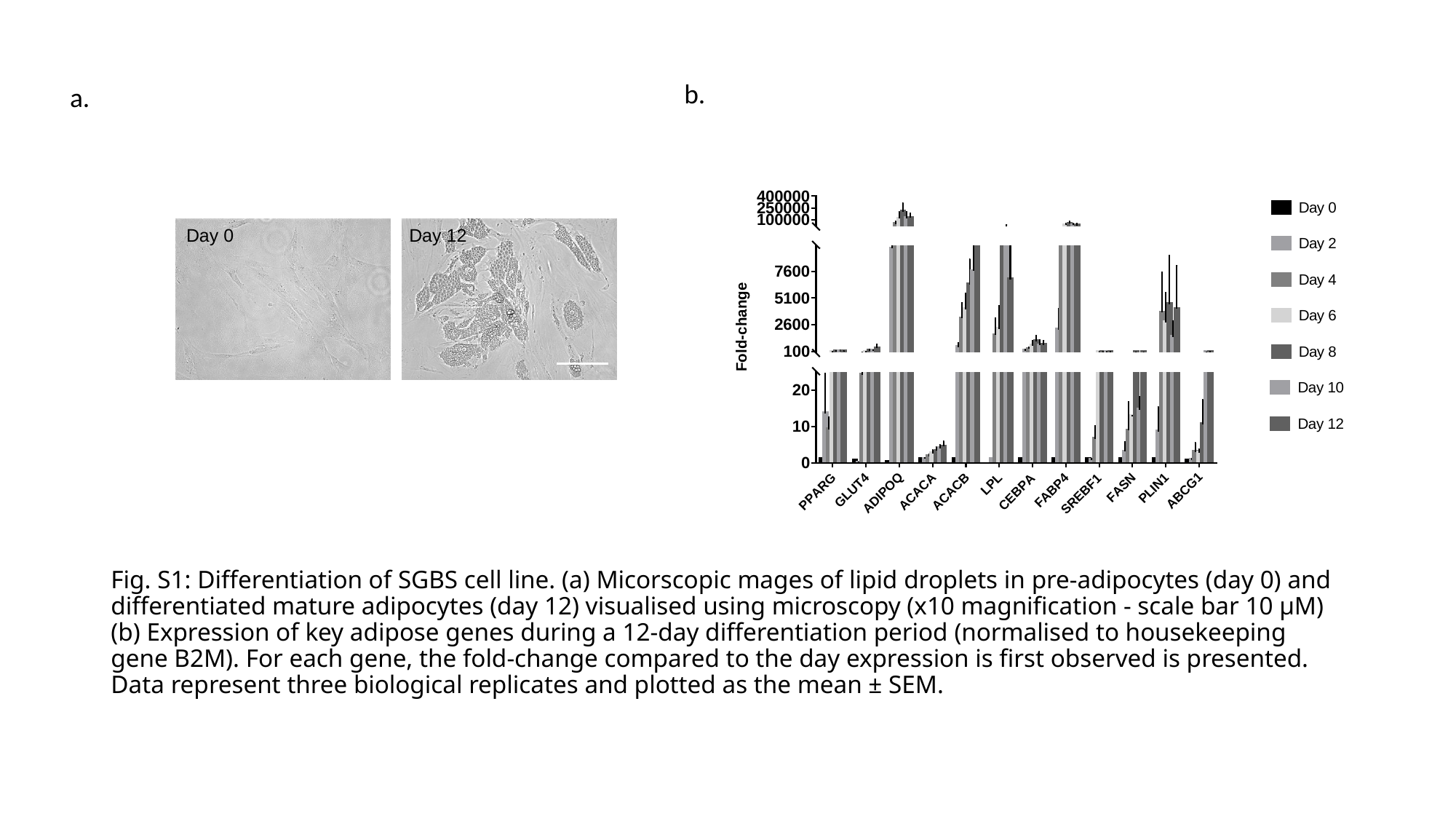

b.
a.
Day 0
Day 12
# Fig. S1: Differentiation of SGBS cell line. (a) Micorscopic mages of lipid droplets in pre-adipocytes (day 0) and differentiated mature adipocytes (day 12) visualised using microscopy (x10 magnification - scale bar 10 µM) (b) Expression of key adipose genes during a 12-day differentiation period (normalised to housekeeping gene B2M). For each gene, the fold-change compared to the day expression is first observed is presented. Data represent three biological replicates and plotted as the mean ± SEM.

## Slide 2
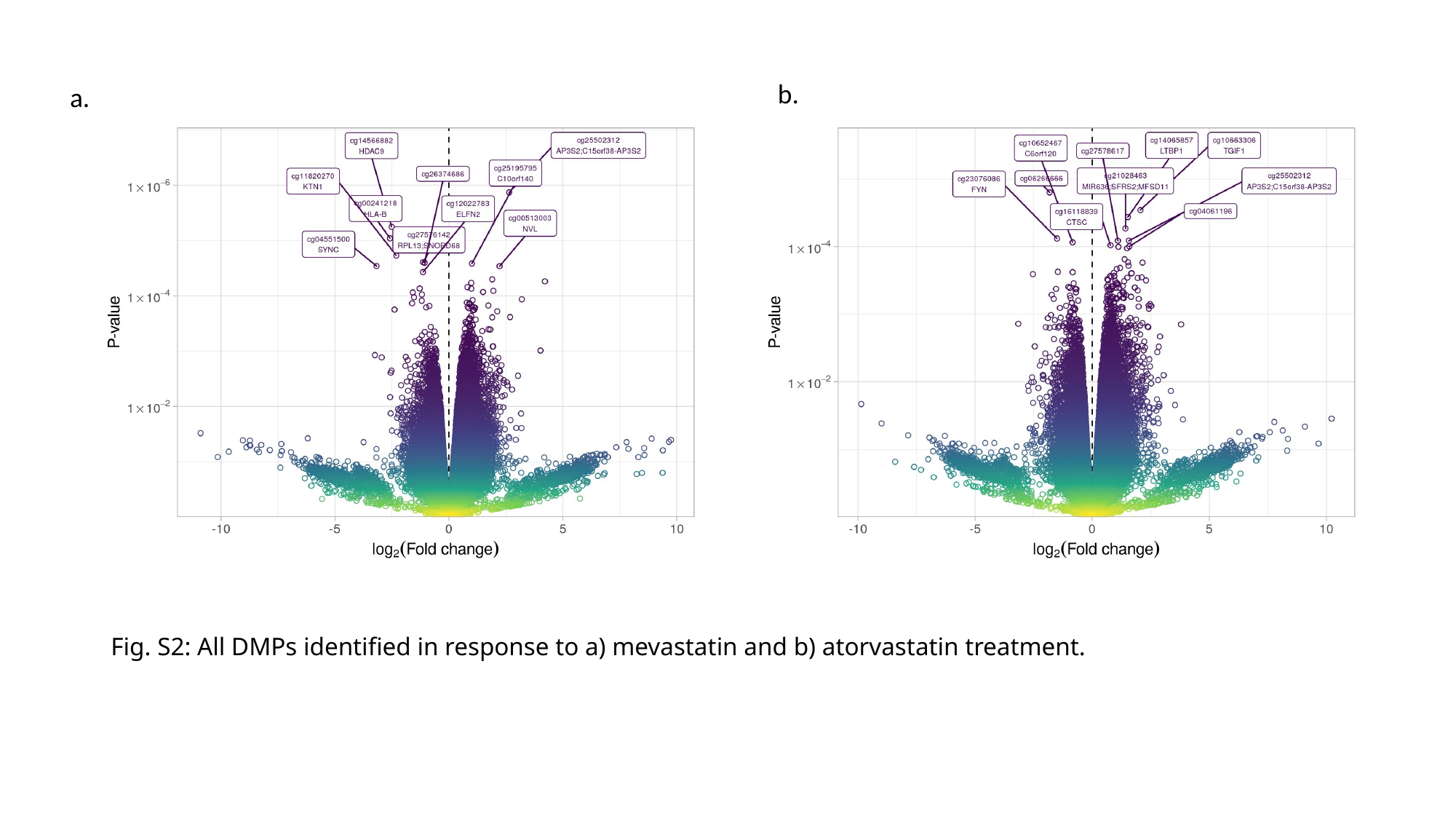

b.
a.
# Fig. S2: All DMPs identified in response to a) mevastatin and b) atorvastatin treatment.

## Slide 3
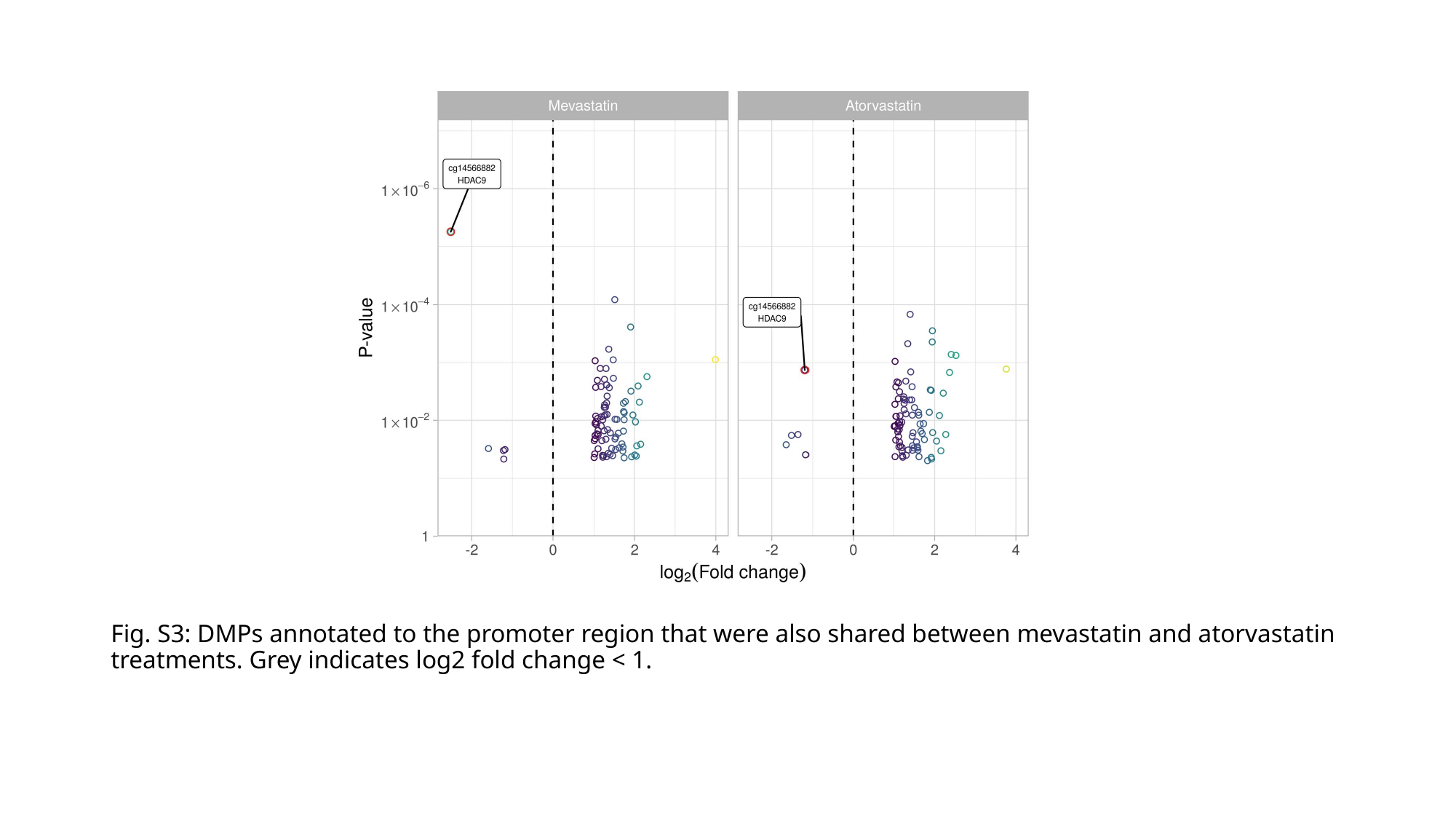

Fig. S3: DMPs annotated to the promoter region that were also shared between mevastatin and atorvastatin treatments. Grey indicates log2 fold change < 1.

## Slide 4
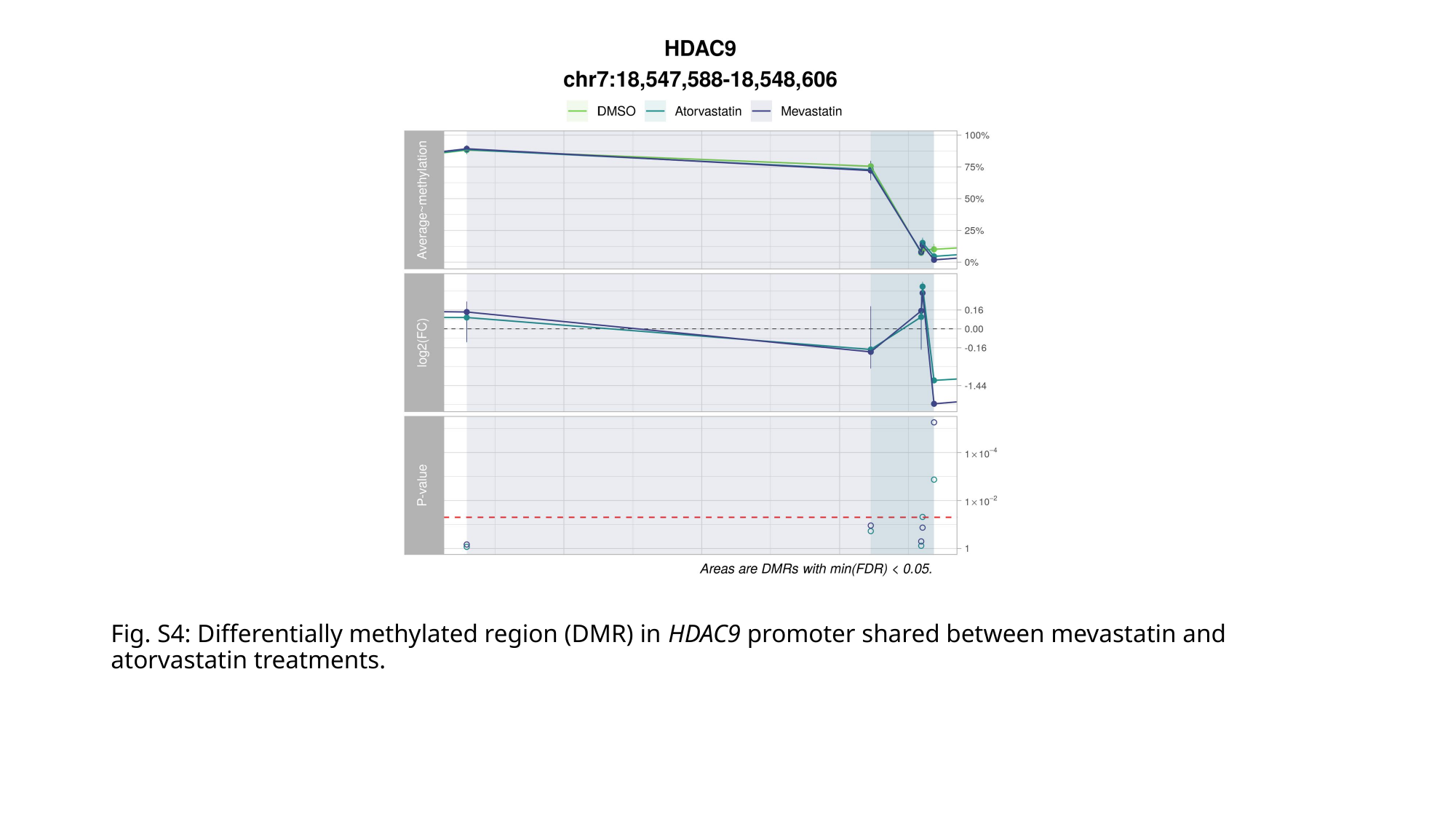

Fig. S4: Differentially methylated region (DMR) in HDAC9 promoter shared between mevastatin and atorvastatin treatments.
